# Assessment of a primary and tertiary care integrated management model for chronic obstructive pulmonary disease

**DOI:** 10.1186/1471-2458-9-68

**Published:** 2009-02-24

**Authors:** Ignasi Bolíbar, Vicente Plaza, Mariantònia Llauger, Ester Amado, Pedro A Antón, Ana Espinosa, Leandra Domínguez, Mar Fraga, Montserrat Freixas, Josep A de la Fuente, Iskra Liguerre, Casimira Medrano, Meritxell Peiro, Mariantònia Pou, Joaquin Sanchis, Ingrid Solanes, Carles Valero, Pepi Valverde, The COPD PROCESS Study Group

**Affiliations:** 1 Department of Clinical Epidemiology and Public Health, Hospital de la Santa Creu i Sant Pau, Autonomous University of Barcelona, CIBER Epidemiology and Public Health (CIBERESP), Barcelona, Spain; 2 Department of Respiratory Medicine, Hospital de la Santa Creu i Sant Pau, Autonomous University of Barcelona, Spain; 3 EAP Encants, CAP Maragall, Catalan Health Institute, Barcelona, Spain; 4 Àmbit Barcelona. Catalan Health Institute, Barcelona, Spain; 5 EAP Xafarinas. Catalan Health Institute, Barcelona, Spain; 6 EAP Dreta de l'Eixample, Barcelona, Spain; 7 SAP Badalona-Sant Adrià, Catalan Health Institute, Badalona, Spain; 8 CAP Sant Andreu, Catalan Health Institute, Barcelona, Spain; 9 EAP Clot, Catalan Health Institute, Barcelona, Spain; 10 SAP Dreta, Catalan Health Institute, Barcelona, Spain; 11 EAP Gaudí (CAP Sagrada Familia), Consorci Sanitari Integral, Barcelona, Spain

## Abstract

**Background:**

The diagnosis and treatment of patients with chronic obstructive pulmonary disease (COPD) in Spain continues to present challenges, and problems are exacerbated when there is a lack of coordinated follow-up between levels of care. This paper sets out the protocol for assessing the impact of an integrated management model for the care of patients with COPD. The new model will be evaluated in terms of 1) improvement in the rational utilization of health-care services and 2) benefits reflected in improved health status and quality of life for patients.

**Methods/Design:**

A quasi-experimental study of the effectiveness of a COPD management model called COPD PROCESS. The patients in the study cohorts will be residents of neighborhoods served by two referral hospitals in Barcelona, Spain. One area comprises the intervention group (n = 32,248 patients) and the other the control group (n = 32,114 patients). The study will include pre- and post-intervention assessment 18 months after the program goes into effect. Analyses will be on two datasets: clinical and administrative data available for all patients, and clinical assessment information for a cohort of 440 patients sampled randomly from the intervention and control areas. The main endpoints will be the hospitalization rates in the two health-care areas and quality-of-life measures in the two cohorts.

**Discussion:**

The COPD PROCESS model foresees the integrated multidisciplinary management of interventions at different levels of the health-care system through coordinated routine clinical practice. It will put into practice diagnostic and treatment procedures that are based on current evidence, multidisciplinary consensus, and efficient use of available resources. Care pathways in this model are defined in terms of patient characteristics, level of disease severity and the presence or absence of exacerbation. The protocol covers the full range of care from primary prevention to treatment of complex cases.

## Background

Chronic obstructive pulmonary disease (COPD), one of the most common diseases in developed countries, has become even more prevalent in recent years. In the Spanish population aged between 40 and 69 years the prevalence is 9.1% (95% confidence interval 8.1%–10.2%) overall, with rates of 14.3% among men and 3.9% among women [1]. The prevalence is higher in men and in older age groups, exceeding 20% in males over the age of 64 years [1]. Rates vary by geographic area, ranging between 4.9% and 18% [2]. COPD has a profound impact on health and quality of life, particularly in periods of exacerbation or acute illness [3]. Given the high prevalence of COPD in the economically active population in addition to reports that an estimated 37% of patients experience limitations in activities of daily living, this disease generates considerable absenteeism and disability [4].

COPD and exacerbations overload the health-care system, accounting for 10% of visits to respiratory medicine specialists and 10% to 12% of primary care consultations [5]. This disease is the third most common reason for hospital admission (2.5%) and leads to an average stay of 8.4 days [6]. Over the course of a year, 12.8% of patients with COPD require hospitalization, 13.8% require emergency care, and 23.8% visit the doctor monthly [7], generating an average of €198.17 in direct medical costs per new diagnosis of COPD per year [5]. The average rises to over €900 per year for previously diagnosed cases [5]. COPD is the fourth leading cause of death in developed countries, preceded only by cancer and cardio- and cerebrovascular diseases [8]. Projections indicate that it will be the third leading cause of death worldwide in 2020 and also one of the main causes of disability and years of life lost [9].

The magnitude and seriousness of the problem contrast with the fact that COPD is a preventable, treatable and modifiable disease. COPD begins to develop earlier than is usually assumed and recognition generally comes when the disease is already in advanced stages. The rate of underdiagnosis is alarming. One population-based study found that 78.2% of cases identified had not been previously diagnosed [2]. The reasons for this are not well understood, but many agree that inadequate coordination between primary and specialized care may play an important role [10]. Health-care systems have long been characterized by a lack of cohesion between these two levels, and even today, no optimal model has emerged to bridge the gap. Spanish outpatient care has been reformed in recent years, leading to substantial primary health-care improvements in the public sector. However, the nature of relations between primary care physicians and specialists has not been a central concern, contributing to the gap between the two levels of care and to mutual lack of understanding. This is of particular importance with regard to COPD given its high prevalence and wide range of severity in the patient population. The first attempts at writing agreements on care for COPD patients were undertaken by scientific societies only a few years ago [11], but clear protocols to guide relations between levels of care, validated to demonstrate their effectiveness and efficiency, are still lacking. Nor are there studies on which to base the recommendation of one protocol over another.

A model for managing the care of COPD patients is still needed. The aim of such a model would be to integrate available health-care resources, helping the various clinicians involved to agree on a common approach and act in concert. An ideal, shared model would facilitate cooperation among health-care staff on the basis of well-defined responsibilities, agreement on working protocols and mutual exchange of information. It would serve to promote effective health-care for all patients as well as the efficient use of resources.

There is relatively little experience with COPD management models in different countries and few have been fully evaluated. The considerable heterogeneity of interventions applied makes comparison difficult, and most models also suffer from limited scope, addressing certain aspects of the disease rather than encompassing prevention as well as care in both stable and exacerbated phases. Reported models also fail to bring together practices in the different health-care settings in which professionals work inside and outside hospitals.

Most studied programs focus on pulmonary rehabilitation. In this respect, although the National Emphysema Therapy Trial in California, for example, was unable to demonstrate improvements either in mortality or exercise tolerance [12], others have managed to achieve benefits with home- or hospital-based programs. One study found that exercise tolerance increased, dyspnea decreased and hospital use declined [13]; another reported that patients achieved better quality of life on general and respiratory-disease measures [14].

Models that include patient education for self-management of COPD, applying various types of supervision and support, are also noteworthy in the literature. They have been shown to produce a long-term effect on hospital admissions, visits to emergency rooms and sporadic visits to the doctor [15,16] as well as on quality of life [17]. The most recent systematic reviews on this topic seem to confirm a reduction in hospital admissions but because of the great heterogeneity of interventions and outcome measures assessed, it is still not possible to make recommendations on the manner and content of self-management training [18,19].

Other care models are based on the multidisciplinary development, implementation and assessment of clinical practice guidelines that include tools to support clinical decision making. Such practice guidelines have been associated with a shift away from emergency admissions toward more planned, scheduled care [20]. The evaluation of health outcomes has shown that quality-of-life scores on respiratory-disease-specific questionnaires improve [21] and that there is a moderate positive effect on lung function [22].

Finally, a few studies have evaluated programs for integrated care, the so-called disease management programs. These are implemented in specific geographic locations, are population-based, coordinate different levels of health care, and offer integrated care, meaning that they encompass interventions related to all stages in the natural history of the disease. In COPD, these programs have had a positive impact on certain aspects of patient quality of life as well as on reducing the number of hospital admissions and the length of hospital stays [23-27].

In spite of experiences described in the international literature, however, we still know little of those models and their impact on COPD patients' health. Nonetheless, the available evidence does indicate that it is feasible to develop more cost-effective systems for providing health care, and that these frameworks have the potential to provide greater health benefits.

In Spain, several programs, all fragmentary in their scope, have used markedly diverse approaches and interventions. For example, one group who evaluated the short- and long-term effects of a pulmonary rehabilitation program in a randomized clinical trial, found significant benefits on perceived breathlessness, on 6-minute walk test results, on several domains of the Chronic Respiratory Questionnaire (CRQ), and on the number of exacerbations [28]. Hospital admissions, however, were not reduced. Another trial on pulmonary rehabilitation is being carried out in 16 primary health-care centers in the autonomous community of Madrid; its results are not yet published [29]. Yet another group of researchers, in the PADOC Project, tested a program to increase the rate of diagnosis of COPD [30]. Targeting undiagnosed individuals with risk factors, the program was implemented in the primary care setting under the supervision of a large teaching hospital. Spirometry was performed by 194 primary care physicians and later confirmed by respiratory medicine specialists at the referral hospital. The diagnosis of COPD was confirmed in 55% of the cases in that trial. Various Spanish programs have sought to reduce admissions and shorten hospital stays for exacerbations. A program in the Basque Country sent a nurse specialist to the homes of patients who were discharged in less than 4 days [31]. The CHRONIC program in Barcelona tested a protocol for the coordination of primary care resources available to patients after discharge [32-34]. That program provided a nurse case manager and relied on telephone support for patients. Both programs led to significant reduction in the use of hospital services in the test groups compared to the control groups, with regard to admissions, readmissions, and duration of hospital stay. Patient satisfaction was also greater in the intervention group. The PRICE program for the integrated care of patients with COPD, currently being implemented by respiratory medicine and primary care physicians in the autonomous community of Madrid, is also noteworthy for its combination of all the characteristics required of a clinical management system [35]. PRICE defines a multidisciplinary framework for health care, coordinating all care levels, and provides unified guidelines for all professionals involved. The aim is to improve patient care and achieve more rational use of available resources. Finally, the AUDIPOC project is now under way [36]. This multicenter study in 7 Spanish autonomous communities aims to improve the quality and effectiveness of clinical care given to patients with exacerbated COPD; the method is based on nationwide clinical audits and the establishment of guidelines for clinical management of exacerbations.

At this time, the COPD PROCESS protocol is being implemented by Hospital de la Santa Creu i Sant Pau in Barcelona in conjunction with the primary care teams this hospital serves. PROCESS is based on the assumption that the patient is the center and origin of all health-care activities intended to prevent or address his or her clinical manifestations. In so doing, PROCESS applies an ambitious management model that brings together both physicians and nurses in all the medical specialties that are relevant to the treatment of COPD. A series of multidisciplinary interventions have been established with a view to integrating the various aspects of the disease and coordinating levels of care. The model attempts to offer a practical, useful tool for patient care and to avoid becoming yet another expert consensus statement with scarce following and negligible impact.

This paper sets out the means through which the effectiveness of this clinical management model will be assessed. The overall purpose of the evaluation is to provide evidence on the degree of control of the disease achieved and on the improvements in the management of patient care, as well as to determine the impact on health outcomes and quality of life. PROCESS is intended 1) to improve patient care delivery, particularly through rational use of primary and specialist care, and 2) to promote health and quality of life, both in comparison with the conventional health-care delivery system in the same city. The principal endpoints of the study will be the rate of hospital admissions and quality of life.

The hypothesis to be tested is that the PROCESS model provides effective health-care management that uses available resources more efficiently and that greater clinical benefit comes as a result. If we confirm this hypothesis, we will make available a health-care model of demonstrated effectiveness, from which we will be able to contribute to improving the quality of care given to patients with COPD.

## Methods/Design

**Figure 1:**
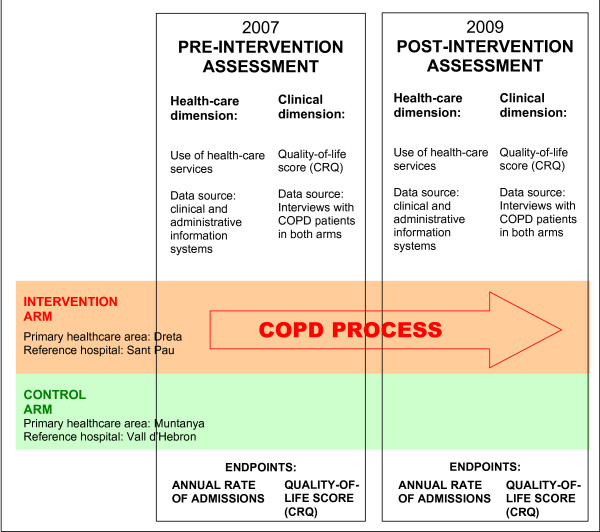
**Design and evaluation of the COPD PROCESS model, a multidisciplinary disease management program for chronic obstructive pulmonary disease**. COPD indicates chronic obstructive pulmonary disease.

A quasi-experimental design based on comparison of two non-randomized groups was chosen to assess the effectiveness of the multidisciplinary, multicenter COPD PROCESS management model. The intervention group will consist of all patients with COPD in the area served by Hospital de la Santa Creu i Sant Pau in Barcelona. The control group will comprise all COPD patients in the area served by Hospital de la Vall d'Hebron, also in Barcelona. Randomization was not feasible because only health-care professionals within the intervention area can undertake the coordinated care protocols necessary for implementing the PROCESS protocol. The study will include pre- and post-intervention assessment after 18 months (Figure 1). This project and the study were presented to and approved by the Clinical Research Ethics Committee of Hospital de la Santa Creu i Sant Pau.

### Setting

The study will be carried out in the patient populations registered with primary care centers within the areas assigned to the respective referral hospitals. The study group will come from 17 primary care centers, of which 10 are managed by the Catalan Health Institute (ICS) and 7 by other types of health-care provider (private for-profit, private nonprofit, and mixed public-private entities). The population base was 322,478 inhabitants over the age of 14 years. The prevalence of COPD in this population was estimated by age and gender from the results of the IBERPOC study [2] and the Catalan Health Survey of 2002 [37]; based on those studies, we expect 32,114 COPD patients (prevalence 8.18%). The control group will also come from a health-care area comprising 17 primary care centers, all of which pertain to the ICS (307,668 inhabitants over the age of 14 years; 32,248 patients).

### Type of participant

All patients over 20 years old whose primary health-care medical records included any of the international disease classification codes related to COPD (Table 1) will be considered COPD patients and candidates for enrollment. Diagnostic codes for chronic obstructive asthma (with COPD), bronchiectasis, extrinsic allergic alveolitis, and respiratory failure will be excluded. Given that there are records for individuals classified as having COPD but who cannot be located for a variety of reasons (for example, change of address or change or primary care clinic), we will include only those who have attended a medical visit or picked up prescriptions within the past year. All patients so-diagnosed with COPD will be included in both the intervention and control areas.

**Table 1:** International disease classification codes for chronic obstructive pulmonary disease on which patient inclusion will be based.

**ICD-10**	**ICD-9**	**ICPC**
**J41 Simple and mucopurulent chronic bronchitis**	**490 Bronchitis, not specified as acute or chronic**	**R79 Chronic bronchitis**
J41.0 Simple chronic bronchitis	**491 Chronic bronchitis**	
J41.1 Mucopurulent chronic bronchitis	491.0 Simple chronic bronchitis	
J41.8 Mixed simple and mucopurulent chronic bronchitis	491.1 Mucopurulent chronic bronchitis	
	491.2 Obstructive chronic bronchitis	
	491.20 Obstructive chronic bronchitis – without exacerbation with the 2004 code modification: *Emphysema with chronic bronchitis – without exacerbation*	

**J42 Unspecified chronic bronchitis**	**491.9 Unspecified chronic bronchitis**	

**J43 Emphysema**	**492 Emphysema**	
J43.0 MacLeod's syndrome	492.0 Emphysematous bleb	
J43.1 Panlobular emphysema	492.8 Other emphysema	
J43.2 Centrilobular emphysema		
J43.8 Other emphysema		
J43.9 Emphysema, unspecified		

**J44 Other chronic obstructive pulmonary disease**	**491.21 Obstructive chronic bronchitis with acute exacerbation **with the 2004 code modifications:	**R95 COPD **(chronic obstructive pulmonary disease)
J44.0 Chronic obstructive pulmonary disease with acute lower respiratory infection	*Acute bronchitis with chronic obstructive pulmonary disease (COPD)*	
J44.1 Chronic obstructive pulmonary disease with acute exacerbation, unspecified	*Acute and chronic obstructive bronchitis*	
J44.8 Other specified chronic obstructive pulmonary disease	*Emphysema with acute and chronic bronchitis*	
J44.9 Chronic obstructive pulmonary disease, unspecified	*Acute exacerbation of chronic obstructive pulmonary disease (COPD)*	
	*Excluded: chronic obstructive asthma with acute exacerbation (493.22)*	
	**491.8 Other chronic bronchitis**	
	**496 Chronic airway obstruction, not elsewhere classified**	

ICD = International Classification of Diseases, with modifications approved by the World Health Organization

ICPC = International Classification of Primary Care (WONCA – World Organization of Family Doctors)

Additionally, a cohort will be selected in order to analyze the clinical and health-care variables. These patients must also meet the following additional inclusion criteria: 1) be able to perform spirometry; 2) meet spirometry criteria for a diagnosis of COPD: lung disease characterized by irreversible airflow limitation defined by a ratio of forced expiratory volume in 1 second [FEV_1_] to forced vital capacity ≤70% after a negative bronchodilator test, and in case of a positive postbronchodilator test (an increase in FEV_1 _of >12% and >200 mL after the use of 400 μg of salbutamol) not having a personal history of asthma, symptoms of atopy or a smoking habit of <40 pack-years. Additional exclusion criteria for this cohort were 1) a diagnosis of any terminal disease that indicates a survival prognosis <6 months, 4) advanced heart failure, 5) sequelae of pulmonary tuberculosis, and 6) lung cancer. All patients will have to give their written, informed consent to be enrolled.

Patients with other respiratory or nonrespiratory diseases can be included in the study provided they do not interfere with measurements. The testing of patients in a period of COPD exacerbation will generally be postponed until the disease has been stable for at least one month. Patients with exacerbated disease will not be excluded from the study, however; in case of continuously recurring exacerbations, the patient will be tested in that clinical state.

Calculation of the cohort size: For two-tailed comparisons, accepting an alpha risk of 0.05 and a beta risk of 0.2, to detect a statistically significant between-group CRQ score increment of 0.5 points (the minimum clinically significant difference) [38], and assuming a homogeneous standard deviation of 1.7 and a loss to follow-up of 10%, we have calculated that 220 patients will be required in each cohort. A simple random sampling procedure will be used to select the cohort patients.

### The COPD management model

**Figure 2:**
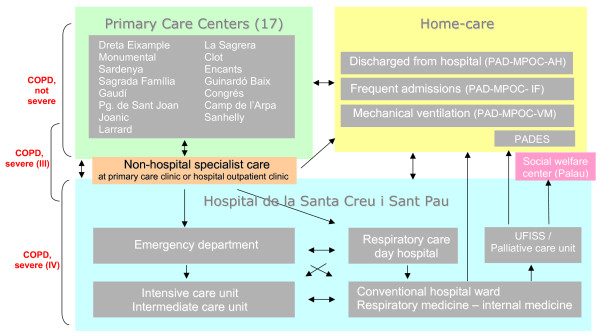
**Participating centers and the care pathways of the COPD PROCESS model**. COPD indicates chronic obstructive pulmonary disease.

The COPD PROCESS system was conceived through consensus among all the health-care professionals at different levels of the system; this included agreement on medications between community and primary-care pharmacists. A description of the project has been published [39] and a series of brochures have provided a summary. Clinical practice protocols for each phase of the disease have been defined in accordance with the recommendations of the Global Initiative for Chronic Obstructive Lung Disease (GOLD) [40] and two Spanish national guidelines: the most recent version of the recommendations of the Spanish Society of Pulmonology and Thoracic Surgery (SEPAR) [41] and the consensus paper of the Spanish Society of Family and Community Medicine (semFYC) [42]. The result is a set of algorithms and care pathways reflecting essential diagnostic and therapeutic protocols that have been adapted to conditions in the local health-care system. PROCESS proposes a clinical management program for COPD that places the patient at its center and integrates care within routine clinical practices at different levels. Resources are organized to provide the best possible care and treatment through coordinated multidisciplinary actions (Figure 2).

Clinical recommendations under the COPD PROCESS model are defined in accordance with patient characteristics and level of disease severity, and the presence or absence of exacerbation. The recommendations, which are not dependent on the setting where health care is delivered, are grouped as follows: 1) prevention (general and specific interventions that target smoking dependency); 2) diagnosis and determination of severity based on GOLD criteria [39]; 3) education (basic information for the patient with COPD); 4) monitoring (periodic follow-up for the patient whose disease is stable and not severe, or stable and severe, or very severe); 5) treatment of exacerbated non-severe and severe COPD; and 6) home care for the COPD patient by means of three programs. The first (the PAD-MPOC-AH program) addresses the needs of the discharged patient. In this program, the primary care team receives discharged patients within the first 72 hours after they arrive home. The second (PAD-MPOC-IF) is designed to manage situations of frequent admission by bringing together primary and specialist health-care professionals in a committee to take charge of the treatment and follow-up of frequently-admitted patients. The third program (PAD-MPOC-VM) will address the needs of patients receiving home noninvasive ventilation.

Each set of recommendations in the PROCESS management model defines interventions and care in the following categories: 1) medical history, physical examination, and diagnostic tests; 2) pharmacologic and non-pharmacologic therapies (smoking cessation, pulmonary rehabilitation, nutritional, education, oxygen therapy, and noninvasive ventilation); and 3) follow-up that is coordinated between the levels of care in accordance with a model of mixed consultation and direct care, including criteria for referral, admission to a conventional hospital ward or treatment in intensive or specialized intermediate care units.

The implementation of the model rests on five types of action that have been undertaken in the preliminary phase. The first involves the formation of working groups and the identification at each primary care center of two referees, a physician and a nurse, who will be responsible for the program at that facility. Second, the physicians and nurses will attend informative sessions at the hospital and primary care center. These sessions will explain the clinical practice protocols and care pathways in the PROCESS management program. This action will also involve explaining the project to the health-care facilities' administrators and managers; official institutional approval and involvement will thereby be assured. The third will involve professional development and training through 1) a session in each primary care center, 2) training for a group of nurses, and 3) the preparation of patient educational support materials. The fourth set of activities will investigate the manner of performing spirometry at each primary care center. Based on the findings, the primary and tertiary care staffs will develop systems for calibrating equipment and assuring the quality of spirometries Training sessions will be set up to show primary care physicians and nurses how to perform and interpret spirometry. The fifth preliminary-phase action will be the introduction of a software application into the medical records of the primary health-care teams. This application will support clinical decision-making in accordance with the PROCESS protocols. This software is currently being developed by the ICS.

These five preparatory actions were begun in 2006. The preliminary phase is scheduled to be completed by the end of 2008, after which the clinical management program will be implemented in its entirety. None of the aforementioned actions will take place in the centers located in the control area of the city's health-care system. Nor will the clinical and organizational interventions be applied there. The care of COPD patients in the control area will continue according to the usual practices of physicians in those centers.

### Study variables

Table 2 shows the variables measured for the study [43-46]. Two complementary dimensions of the PROCESS model will be measured. One involves health-care management. To assess that dimension, clinical and administrative data for the entire population will be extracted from the records of the participating centers. The inclusion of this dimension addresses the issue of the impact on the health-care system (objective 1). The second, clinical dimension will be evaluated on the basis of data from the intervention and control cohorts. For that purpose, data will come from both medical records and visits with patients. The assessment of this dimension corresponds to the evaluation of patient health and quality-of-life benefits (objective 2).

**Table 2:** Study variables for the evaluation of the COPD PROCESS

**Group**	**Variables**
Social and demographic characteristics:^†‡^	Primary care clinic assignment; date of birth; sex.
Patient and lung function characteristics:^‡^	Weight; height; spirometry performed in the past year.
	Spirometry results after bronchodilator (Datospir 120, model A, SIBEL, S.A, Barcelona, Spain; after calibration, with daily maintenance by expert nurses, and quality assurance).
	Carbon monoxide in expired air; pulse oximetry; COPD stage (GOLD criteria); year COPD was diagnosed.
Clinical status (last 6 months):^†‡^	Smoking (frequency, pack-years, cessation phase, enrollment in a cessation program, adherence to recommended preventive measures); exercise (type of activity, duration in minutes and days per week); adherence to any medical advice that has been given; Charlson comorbidity index [43]; dyspnea severity (Medical Research Council Breathlessness scale [44]).
Medication:^‡^	Active principle and pharmaceutical class, dosage.
	Inhalers: number of inhaler devices used in the last month, level of handling expertise (correct performance of 3 actions for a specific inhaler type).
	Oral medications (glucocorticosteroids, theophylline, antioxidants) and their agreement between the patient's report and the medical record.
	Supplemental oxygen therapy; mechanical ventilation; annual vaccination or in the previous winter (flu, pneumococcus)
	Attendance at COPD management training sessions or specific sessions related to medication type.
	Deviations from the PROCESS model (lack of agreement between drugs taken and those recommended for the recorded GOLD severity stage).
Exacerbations in the last year:^†‡^	Defined as the worsening of respiratory symptoms requiring treatment with antibiotics or oral corticosteroids or both (moderate exacerbation) or hospitalization (severe exacerbation) or a combination of the two degrees of severity (based on [45])
Complications and potential complications:^‡^	Primary care nursing diagnosis (according to the Catalan Health Institute's adaptation of the North American Nursing Diagnosis Association (NANDA), February 2003).
Health care in the past year:^†‡^	Primary care clinic visits for any reason (including the general practitioner, the nurse or the respiratory medicine specialist); number of home visits made in the past year; number of visits to hospital (outpatient, emergency, admissions).
Quality of life:^‡^	Respiratory-disease related quality of life (Chronic Respiratory Questionnaire [46])
Systemic inflammatory status:^‡^	C-reactive protein (capillary blood levels: QuikRead^® ^CRP 101. Orion Diagnostica, Espoo, Finland)
Other:^†‡^	Exitus

^† ^Measures evaluating the health-care dimension of the COPD PROCESS model. Information will be gathered on the population of patients with chronic obstructive pulmonary disease in each of the two study areas (clinical and administrative data from the databases of the participating centers).

^‡ ^Measures to evaluate the clinical dimension of the COPD PROCESS model. Information will be gathered on patients in the intervention and control cohorts by consulting patient medical records and arranging for patient visits in each of the centers.

All measurements will be recorded at the start of the study and after 18 months' experience implementing the management program.

### Data collection and analysis

Figure 1 shows details of analysis of the pre- and post-intervention data.

The clinical and administrative information systems at the primary care centers are computerized (eCAP in the ICS centers and other applications such as the OMI-AP or SinAPsis in other centers). Primary care physicians in the intervention area will also be using PROCESS software linked to these applications. That software will serve two functions: it will guide decisions to bring them into line with the PROCESS recommendations and will also provide a place to record the actions taken. The participating hospitals also have computerized clinical and administrative information systems for outpatient, emergency, and day-hospital activity as well as admissions. The data will be merged into a single COPD patient registry for each of the areas (intervention and control). Patients will be identified by their personal identification code. Information in this registry will, if possible, be complemented with information from records of prescriptions for outpatient respiratory therapy, from the Minimum Basic Data Set for discharged patients, from records of pharmaceutical prescriptions filled, and from the death registry of the Department of Health of the Government of Catalonia (Generalitat). Finally, data will also be available from the records of an allied social-welfare and health-services program (Centre Palau).

The clinical data of each patient in each cohort will be collected by two nurses experienced with COPD management who will go to the primary care center of each patient. There they will systematically extract information from the medical records (medical history and other documents in digital form or on paper). They will interview each patient to check the inclusion criteria, obtain informed consent, ask questions relevant to the outcome measures, perform lung function tests, and administer a questionnaire about health-related quality of life.

### Statistical analysis

Patients with COPD in the intervention and control areas will first be described using parameters of point estimation and their 95% confidence intervals. The groups will then be compared based on bivariate hypotheses on pre- to post-intervention changes in process and outcome variables. The statistical tests that will be used will depend on the nature of the variable studied.

Analysis of the health-care dimension will focus on the impact of the COPD PROCESS model within the health-care delivery system (objective 1), based on comparing the utilization of resources and visits to primary and specialist care facilities in the intervention and control areas. The principal outcome measure will be the change in annual rate of scheduled and unscheduled admissions that are made with a main diagnostic code of COPD (Table 1) or another code related to a respiratory system process. The year prior to implementation of the PROCESS management system will be compared to the last 12 months of implementation. The difference between the rates of the two study areas will be estimated using the indirect standardization method in which the population assigned to each health-care area will be used as the standard. If possible, a more precise analysis of the differences in rates will be undertaken by constructing linear multiple regression models adjusted for the distribution of the dependent variable (negative binomial, Poisson, beta), a procedure which corrects for confounding factors.

For the clinical dimension, health status and quality of life will be compared between patients in the intervention and control areas (objective 2). The main outcome measure will be minimal clinically significant differences between pre- and post-intervention quality-of-life scores. A test of comparison between two independent means will be used for comparisons with the control group. If multivariate analysis is necessary, multiple regression models will be constructed.

## Discussion

The COPD PROCESS evaluation study offers a unique opportunity to learn about the performance of a health management model that is integrated, multidisciplinary, adapted to conditions in Spain and possibly appropriate for countries with similar characteristics. The knowledge gained will be an important contribution given that few full descriptions of such experiences are available in the literature. If we are able to demonstrate the feasibility and benefits of the PROCESS model, a substantial innovation in health-care organization will become available. Objective, scientific evidence of the impact of a model of health-care delivery on COPD in terms of patient health and quality of life will be of great importance and help palliate the considerable morbidity and mortality this disease causes. The result will be a better system in which resources for patient care are handled more rationally and efficiently to deliver higher quality health care.

Although the fact of implementing the model in a local health-care area may suggest that the results cannot be extrapolated to other settings with different characteristics, the intervention area has no singularly differentiating characteristics and the project will require no investment of resources other than those usually available to any similar health-care system. This project therefore has the external validity necessary to generalize the results to at least the Spanish national health-care system and assure that the impact it might have on health and the health-care system will be highly relevant.

### Strengths and possible limitations of the study

The limitations inherent to any evaluative or quasi-experimental study are also present in this research. Patients cannot be randomized for this study. Nonetheless, the design is the most robust one available and will provide the strongest evidence possible, within the existing constraints. Possible differences that might emerge between the control and intervention groups will be adjusted by applying multiple regression analysis.

The use of clinical and administrative databases as sources of information places limitations on the comprehensiveness of the analysis and its validity, as the quality of data is dependent on the information storage interface and its adequate and proper use by health-care professionals. It was expressly decided, however, not to create dedicated databases given that the pragmatic nature of the model requires that the information sources be integrated into the daily work routines of the physicians and nurses who are caring for the patients. The inclusion of the study cohorts will counterbalance this limitation.

Implementing a multidisciplinary model over such an extensive geographic area and working with such a large number of clinical caregivers is a complex undertaking. Adequate and consistent adherence to the PROCESS protocols at all the health-care facilities will require a very high level of dedication. Therefore, it will be essential to integrate the project-specific software into the records of the care facilities. Periodic meetings to coordinate and supervise the participants will also be required.

## Abbreviations

COPD: Chronic Obstructive Pulmonary Disease; FEV_1_: Forced expiratory volume in 1 second; GOLD: Global Initiative for Chronic Obstructive Lung Disease; ICD: International Classification of Diseases, with modifications approved by the World Health Organization; ICPC: International Classification of Primary Care; WONCA: World Organization of Family Doctors; UFISS: Social and Health Care Interdisciplinary Functional Unit for geriatric patients of the hospital; PADES: Home Care and Support Teams.

## Competing interests

The authors declare that they have no competing interests.

## Authors' contributions

IB, VP, MAL, LD, MMF, MF, IL, CM, MP, MAP, JS, IS, CV and PV have made substantial contributions to the conception and design, to acquisition, analysis and interpretation of data, to drafting the manuscript and revising it critically for important intellectual content and have given final approval of the version to be published. EA, PAA, AE, JA de la F have made substantial contributions to the conception and design, to acquisition, analysis and interpretation of data and have given final approval of the version to be published.

## Pre-publication history

The pre-publication history for this paper can be accessed here:


